# Effects of Immunomodulatory Therapy on the Skin Barrier Function in Patients with Psoriasis Vulgaris

**DOI:** 10.3390/medicina61112070

**Published:** 2025-11-20

**Authors:** Anete Mālkalne, Vanda Bondare-Ansberga, Ilona Hartmane, Ingmārs Mikažāns, Lelde Reinberga

**Affiliations:** 1 Faculty of Medicine, Riga Stradins University, LV-1007 Rīga, Latvia; 2 Department of Doctoral Studies, Riga Stradins University, Riga 1st Hospital, LV-1007 Rīga, Latvia; 3 Riga 1st Hospital, LV-1001 Rīga, Latvia; 4 Department of Dermatology and Venereology, Riga Stradins University, LV-1007 Rīga, Latvia

**Keywords:** psoriasis vulgaris, skin barrier, immunomodulatory therapy, adalimumab

## Abstract

*Background and Objectives*: Psoriasis vulgaris features epidermal barrier dysfunction. *Materials and Methods*: Barrier function changes were prospectively evaluated over 12 weeks during TNF-α inhibition with adalimumab, along with concurrent changes in disease severity and quality of life. Adults with moderate-to-severe plaque psoriasis initiating adalimumab (80 mg loading on day 1; 40 mg every other week thereafter, starting day 8) underwent assessments at baseline and at week 12 (*n* = 9; mean age 44.1 ± 14.9 years, range 20–61). Transepidermal water loss (TEWL; g/m^2^/h) and skin pH were measured at the elbow, lower leg, abdomen, back, and scalp; PASI, BSA, and DLQI were recorded. The measurements were standardized, though room temperature/humidity were not identical between visits. *Results*: The clinical indices improved markedly and TEWL also decreased at all sites—the elbow, lower leg, abdomen, back, and scalp—indicating barrier recovery; in contrast, the pH remained within a mildly acidic range at all sites. Lesion-to-non-lesion conversion occurred, and no site worsened. *Conclusions*: In summary, 12 weeks of adalimumab were associated with a notable clinical improvement and consistent, site-spanning reductions in TEWL, whereas skin surface pH showed no material change. TEWL appears to be a sensitive objective adjunct to clinical indices for monitoring response.

## 1. Introduction

Human skin, the largest organ of the body, performs a wide spectrum of regulatory and defensive roles essential to body integrity [1,2]. The skin barrier capability resides predominantly in the epidermis, primarily in the stratum corneum, preserving skin homeostasis and protecting the human from environmental stressors [1]. Skin surface pH and transepidermal water loss (TEWL) are some of the objective parameters for determining epidermal barrier function [1].

Psoriasis vulgaris is a chronic, immune-mediated disease arising from the interplay of genetic susceptibility and environmental modifiers, and it is recognized as a systemic inflammatory disorder [3,4] that affects approximately 1–3% of the world’s population [3]. Pathological, persistent inflammatory signaling drives keratinocyte hyperproliferation and aberrant epidermal remodeling [2]. Clinically, psoriasis confers a substantial physical and psychosocial burden attributable to painful cutaneous lesions, body image disturbance, and a high comorbidity load [2]. Consistent with its systemic nature, psoriasis is frequently associated with other immune-mediated diseases, including psoriatic arthritis, inflammatory bowel disease, and coronary artery disease [3,4]. In psoriasis, consistent with its pathogenesis, epidermal barrier abnormalities are observed in lesional skin and TEWL is significantly increased; its magnitude correlates positively with clinical lesion severity [3,4]. However, lesional (and, in some reports, non-lesional) psoriatic skin shows a decreased pH versus healthy skin [5].

Psoriasis severity is determined using the Body Surface Area (BSA) and Psoriasis Area and Severity Index (PASI). It integrates the percentage of involvement across four body regions—the head, trunk, upper limbs, and lower limbs—with regional ratings of plaque intensity—erythema, scaling, and induration—scored separately in each region [1].

The current therapeutic options for psoriasis comprise topical agents, conventional systemic therapies, and immunomodulatory therapy [6]. Topical therapy is generally first-line for mild disease, and more often provides short-term control at lesional sites [6]. In patients with recurrent flares—particularly those with moderate-to-severe involvement—systemic agents are indicated [6]. However, long-term administration is often limited by acceptability and the potential for cumulative multisystem toxicities and carcinogenic risk [6]; also, it usually does not provide a long-term therapeutic effect. Biologic therapies represent a targeted strategy with a generally favorable risk–benefit profile in moderate-to-severe psoriasis, as they selectively neutralize key cytokines and thereby attenuate downstream inflammatory pathways [6]. These biologics are large, complex molecules—monoclonal antibodies with receptor fusion proteins—that specifically engage immune mediators or their receptors to interrupt signaling [6]. In a double-blind randomized trial of the IL-23 inhibitor guselkumab, Rousel et al. demonstrated that treatment normalized lesional stratum corneum ceramide profiles and reduced TEWL to levels comparable with healthy controls, whereas a placebo had no such effect [7]. The changes in ceramide composition closely paralleled the improvements in barrier function and clinical severity, underscoring the interdependence between epidermal lipids, TEWL, and psoriatic inflammation [7]. However, TNF-α is a central cytokine in the initiation of plaque psoriasis and contributes to its chronic persistence [6,8]. It mediates pro-inflammatory signaling, augments keratinocyte proliferation, promotes angiogenesis, modulates apoptosis, and drives the recruitment and retention of immune effector cells within lesional skin [6,8]. Anti-TNF-α biologics—etanercept, infliximab, adalimumab, and golimumab—bind and neutralize TNF-α, thereby preventing receptor engagement and attenuating the downstream activation of inflammatory pathways [6]. Although biologic therapies targeting TNF-α effectively reduce inflammation and lesion severity, data on their effects on objective skin barrier parameters are still lacking. Most barrier-focused studies in psoriasis have either concentrated on IL-23 pathway inhibition or a combination of different TNF-α antagonists, and detailed site-specific TEWL and pH dynamics during adalimumab monotherapy remain insufficiently characterized. The clarification of these data could clarify whether clinical improvement parallels physiological barrier restoration.

The objectives of this study were (1) to compare skin homeostasis and barrier function in patients with psoriasis vulgaris before and during treatment with a tumor necrosis factor-α (TNF-α) inhibitor—adalimumab—and (2) to determine whether disease severity and patient-reported well-being improve over the course of therapy.

It was hypothesized that 12 weeks of adalimumab therapy would improve skin barrier integrity in adults with moderate-to-severe psoriasis, reflected by a reduction in transepidermal water loss (TEWL) across all body sites, while skin surface pH would remain within the physiologic acidic range.

## 2. Materials and Methods

This study was designed as a prospective, exploratory pilot study to evaluate preliminary changes in skin barrier function during adalimumab therapy. A small, convenience sample (*n* = 9; 3 females; 6 males; mean age 44.1 ± 14.9 years (range 20–61 years)) was recruited from a single dermatology center to assess feasibility and generate effect-size estimates for future larger studies. Patients with moderate to severe psoriasis vulgaris who initiated immunomodulatory therapy with a TNF-α inhibitor—adalimumab—were included in the study. Patients used the standard scheme for plaque psoriasis—loading dose: 80 mg s/c once on day 1; after that, maintenance: 40 mg s/c every other week, starting day 8 (one week after the loading dose).

Skin barrier function was evaluated by measuring skin surface pH using a pH-meter (Skin-pH-Meter PH 905 Courage+ Khazaka electronic GmbH, Köln, Germany), and transepidermal water loss (TEWL) was measured using a TEWL-meter (Tewameter TM 300 with probe heater PR 100 Courage + Khazaka electronic GmbH, Köln, Germany) before treatment initiation at five body areas—the elbow, lower leg, abdomen, back, and scalp (the skin was cleaned with an alcohol-free product). All TEWL and pH measurements were performed in a controlled indoor environment using the same instruments and operator, who used standardized device positioning and contact pressure to minimize inter-rater variability. Although measurement conditions (room temperature and relative humidity) were not identical between visits, all assessments were conducted under comparable ambient conditions after a short acclimatization period (~15 min) in the same room at a similar time of day. For each anatomical site, three consecutive readings were taken, and the mean value was used for analysis. The investigator adhered to a fixed measurement order across visits to reduce procedural bias.

Psoriasis severity was assessed with the PASI and BSA, while patient well-being was evaluated using the Dermatology Life Quality Index (DLQI). All measurements were repeated after 12 weeks of therapy to evaluate changes in skin barrier function, disease severity, and patient quality of life following immunomodulatory treatment. All obtained data were evaluated and analyzed using IBM SPSS Statistics, Version 29.0.0.0 (241) (IBM Corp., Armonk, NY, USA). Data distribution was evaluated using the Shapiro–Wilk test. Because most variables did not meet normality assumptions and the sample size was small (*n* = 9), nonparametric paired analyses were applied. The Wilcoxon signed-rank test was used for continuous paired comparisons, and McNemar’s test was used for categorical paired data. Two-sided significance was set at α = 0.05.

Ethical approval was obtained from the Rīga Stradiņš University Research Ethics Committee (approval code: 2-PĒK-4/187/2025) and the Riga 1st Hospital Ethics Committee (approval code: 16/2024). Informed consent was obtained from all patients involved in the study.

## 3. Results

**Figure 1:**
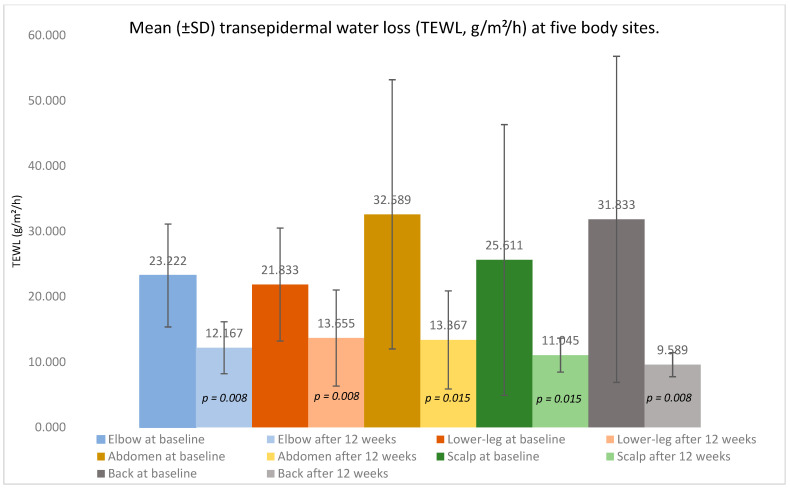
Mean (±SD) transepidermal water loss (TEWL, g/m^2^/h) at five body sites (elbow, lower leg, abdomen, scalp, back) before and after 12 weeks of adalimumab therapy in patients with psoriasis vulgaris.

Across nine adults with moderate-to-severe psoriasis treated with a TNF-α inhibitor, paired analyses (Wilcoxon signed-rank, two-sided α = 0.05) demonstrated a marked clinical improvement over 12 weeks alongside the objective recovery of skin barrier function. TEWL fell consistently at every sampled site—the elbow, lower leg, abdomen, back, and scalp—indicating an improved barrier integrity (Figure 1, Table 1).

Elbow: TEWL declined from 23.222 ± 7.886 g/m^2^/h at baseline to 12.167 ± 3.964 g/m^2^/h (*p* = 0.008). At baseline, the lesional sites showed a higher TEWL (24.522 ± 7.321 g/m^2^/h) than non-lesional sites (12.801 g/m^2^/h); after treatment, the lesional TEWL was 11.623 ± 5.261 g/m^2^/h versus 12.854 ± 1.964 g/m^2^/h in non-lesional skin. Of eight elbows with lesions initially, three converted to non-lesional (37.500% of baseline lesional; 33.333% of patients); McNemar *p* = 0.250.

Lower leg: The mean TEWL decreased from 21.833 ± 8.653 g/m^2^/h to 13.655 ± 7.342 g/m^2^/h (*p* = 0.008). Baseline lesional skin averaged 19.394 ± 8.251 g/m^2^/h versus 30.400 ± 1.412 g/m^2^/h for non-lesional skin; after 12 weeks, the average was 12.873 ± 3.393 g/m^2^/h versus 15.231 ± 13.472 g/m^2^/h. One of seven lesional lower legs (14.3%) became non-lesional; McNemar *p* = 1.000.

Abdomen: The TEWL fell from 32.589 ± 20.608 g/m^2^/h to 13.367 ± 7.497 g/m^2^/h (*p* = 0.015). Baseline values were 51.253 ± 13.801 g/m^2^/h (lesional) and 17.664 ± 8.928 g/m^2^/h (non-lesional); after therapy, the values were 14.051 ± 3.611 g/m^2^/h and 13.175 ± 8.523 g/m^2^/h, respectively. Two of four lesional abdomens (50%) converted to non-lesional; McNemar *p* = 0.500.

Scalp: The mean TEWL reduced from 25.611 ± 20.718 g/m^2^/h to 11.045 ± 2.581 g/m^2^/h (*p* = 0.015). The lesional versus non-lesional TEWL was 20.782 ± 11.702 g/m^2^/h versus 35.273 ± 34.132 g/m^2^/h at baseline and 11.501 ± 1.956 g/m^2^/h versus 10.681 ± 3.182 g/m^2^/h after treatment. Two of six lesional scalps (33.3%) became non-lesional; McNemar *p* = 0.500.

Back: The TEWL decreased from 31.833 ± 24.953 g/m^2^/h to 9.589 ± 1.841 g/m^2^/h (*p* = 0.008). At baseline, the lesional TEWL was 47.574 ± 24.544 g/m^2^/h versus 23.971 ± 23.077 g/m^2^/h for non-lesional skin; after therapy, the values were 10.101 g/m^2^/h versus 9.533 ± 1.967 g/m^2^/h. Two of three lesional backs (66.7%) converted to non-lesional; McNemar *p* = 0.500.

Across all five anatomical regions, the TEWL decreased consistently, and several sites demonstrated lesion-to-non-lesion conversion without any worsening.

**Figure 2:**
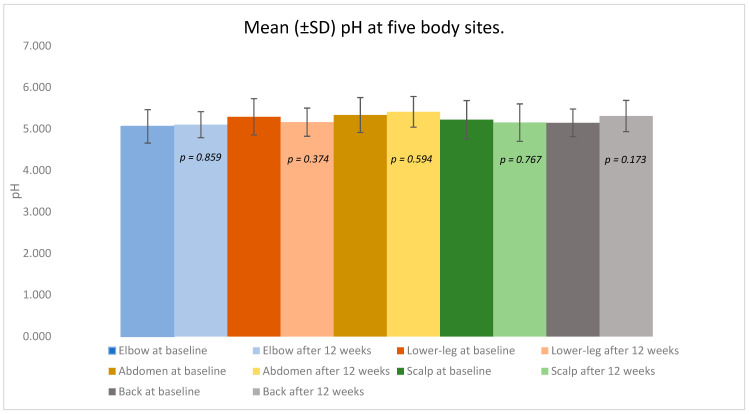
Mean (±SD) pH at five body sites (elbow, lower leg, abdomen, scalp, back) before and after 12 weeks of adalimumab therapy in patients with psoriasis vulgaris.

In contrast, the skin surface pH remained largely stable, showing only small, non-significant shifts around physiologic values across sites (Figure 2, Table 1). The median values moved in the same direction as the means, and the distributional summaries (minimum, maximum, and interpercentile ranges) corroborated these overall trends. Lesion status changes were evaluated with the McNemar test for paired binaries (two-sided α = 0.05).

Elbow: The mean pH changed minimally from 5.061 ± 0.403 at baseline to 5.102 ± 0.314 after treatment (*p* = 0.859). The baseline lesional and non-lesional values were 5.072 ± 0.434 and 4.971, respectively; after 12 weeks, the values were 5.151 ± 0.283 and 5.041 ± 0.395.

Lower leg: The pH remained essentially unchanged, from 5.291 ± 0.439 to 5.162 ± 0.340 (*p* = 0.374). Lesional skin averaged 5.291 ± 0.439 versus 5.301 ± 0.333 in non-lesional areas at baseline, and 5.172 ± 0.421 versus 5.145 ± 0.141 after treatment.

Abdomen: The mean pH shifted slightly from 5.334 ± 0.421 to 5.411 ± 0.370 (*p* = 0.594). The lesional and non-lesional baseline values were 5.221 ± 0.445 and 5.421 ± 0.432, respectively; the post-treatment values were 5.681 ± 0.182 and 5.334 ± 0.381.

Scalp: pH values were stable: 5.222 ± 0.461 at baseline and 5.152 ± 0.451 after 12 weeks (*p* = 0.767). Lesional skin showed 5.141 ± 0.221 versus 5.403 ± 0.811 in non-lesional areas at baseline, and 5.281 ± 0.267 versus 5.058 ± 0.572 after treatment.

Back: The mean pH increased slightly from 5.146 ± 0.334 to 5.310 ± 0.376 (*p* = 0.173). Baseline lesional and non-lesional readings were 5.042 ± 0.301 and 5.205 ± 0.377, respectively; after treatment, the values were 5.281 and 5.314 ± 0.405.

Collectively, the pH values across all examined regions remained within the physiologic acidic range throughout the study, showing no significant deviations or site-specific trends.

**Figure 3:**
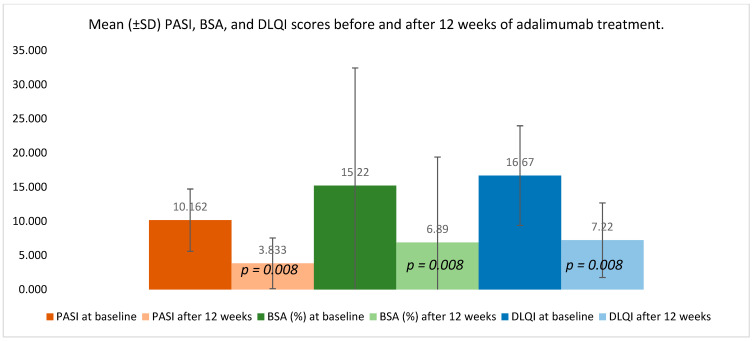
Changes in PASI, BSA, and DLQI after 12 weeks of adalimumab therapy in patients with psoriasis vulgaris.

Clinical indices demonstrated a substantial improvement following 12 weeks of adalimumab therapy (Figure 3). PASI decreased from 10.162 ± 4.553 at baseline to 3.833 ± 3.715 after treatment (*p* = 0.008), reflecting a marked reduction in overall disease activity. BSA involvement declined from 15.220 ± 17.210% to 6.890 ± 12.504% (*p* = 0.008), indicating a meaningful contraction of the affected skin area.

Patient-reported outcomes improved in parallel: DLQI dropped from 16.670 ± 7.297 to 7.220 ± 5.449 (*p* = 0.008), corresponding to a clinically meaningful enhancement in perceived quality of life. The consistent direction and magnitude of these changes confirm the therapeutic response across both objective and subjective measures.

## 4. Discussion

In this single-arm pre–post study of nine adults with moderate-to-severe plaque psoriasis treated with adalimumab, concordant improvements were observed in clinical severity (PASI, BSA) and quality of life (DLQI), together with a consistent reduction in TEWL across all sampled anatomical regions over 12 weeks. However, the skin surface pH remained largely stable within the physiologic acidic range. These findings support the view that systemic TNF-α inhibition not only reduces the clinical burden of psoriasis but is accompanied by a measurable recovery of epidermal barrier function at lesional sites.

TEWL is a direct, functional index of stratum–corneum permeability. The downward shift and range contraction—most pronounced on the back and scalp—indicate an improved barrier integrity during treatment. Although the absolute reductions in TEWL may appear modest, prior studies indicate that even small decreases can represent a meaningful restoration of barrier integrity when accompanied by a consistent clinical improvement. In psoriasis, lesional TEWL values typically exceed normal levels by several-fold, and downward shifts of 5–10 g/m^2^/h toward physiologic ranges have been interpreted as indicative of barrier recovery rather than random fluctuation. In this study, TEWL reductions were consistent across all measured sites. Pathophysiologically, dampening TNF-driven inflammation reduces keratinocyte hyperproliferation and normalizes differentiation, facilitating the restoration of intercellular lipid lamellae and the cornified envelope; collectively, these changes decrease passive water vapor flux and thus TEWL [6,8]. The parallel improvements in PASI/BSA/DLQI with TEWL strengthen the biological plausibility that barrier repair follows disease control under cytokine blockades.

Compared with our 12-week, single-arm adalimumab cohort—where the TEWL fell consistently at lesional sites across all regions and did not show a systematic rise in non-lesional skin, while surface pH remained stable—Gualdi et al. evaluated etanercept with TEWL measured on a target plaque and adjacent uninvolved skin before treatment and after 6 months under tightly controlled conditions (temperature ≈ 21 °C, humidity ≈ 41%; three replicates per site) [3]. They similarly observed barrier restoration in plaques (TEWL reduction toward healthy values), but reported a paradoxical increase in TEWL in uninvolved skin in all patients (to ~16.7 g/m^2^/h on average), prompting a recommendation for concomitant emollient therapy during anti-TNF treatment [3]. Also, our observations of a reduced TEWL after TNF-α inhibition are in line with recent data on IL-23 pathway blockades [7]. Guselkumab therapy not only improved clinical scores but also normalized lesional TEWL and stratum corneum ceramide profiles to those of healthy controls, whereas a placebo had no effect [7]. Together with our findings, this suggests that different biologic classes—targeting either TNF-α or IL-23—may converge on the restoration of barrier function, through partially distinct immunologic and lipid-metabolic pathways [3,7]. The IL-23/IL-17 axis plays a central role in maintaining psoriatic inflammation by promoting keratinocyte hyperproliferation and suppressing terminal differentiation, which collectively impair the skin’s barrier function [9]. In parallel, both TNF-α and IL-23/IL-17 signaling disrupt stratum corneum lipid organization, including ceramide composition, further aggravating barrier dysfunction [3,7,10]. The suppression of these pathways is therefore expected to reduce cytokine-driven barrier perturbation, allowing a partial normalization of lipid organization and water-handling properties, reflected by the lower TEWL [3,7,10].

However, our data do not reproduce the uninvolved skin TEWL rise seen with etanercept. This discrepancy could reflect drug-specific effects (adalimumab vs. etanercept), exposure duration (12 weeks vs. 6 months), anatomical sampling strategy (multiple body sites in our study vs. one plaque and adjacent skin), and sample size, as well as the known environmental sensitivity of TEWL despite standardization. The differences may also relate to distinct pharmacologic and molecular properties: adalimumab, a full monoclonal antibody, binds both soluble and transmembrane TNF, whereas etanercept is a soluble receptor fusion protein with primarily systemic neutralization [11]. Moreover, findings from the guselkumab study suggest that IL-23 inhibition can also normalize TEWL and stratum corneum lipids toward healthy profiles, supporting the broader concept that different biologic classes may promote barrier recovery through pathway-specific mechanisms [7]. Future controlled head-to-head studies should determine whether these mechanistic and pharmacologic differences account for the variability in TEWL responses across biologics.

In contrast to TEWL, the surface pH showed small, non-significant changes and remained within a narrow acidic band. Several factors may account for this: pH is influenced by the local microenvironment (sweat/sebum, hair-bearing skin). Healthy skin maintains a mildly acidic “acid mantle,” with a natural average around pH 4.7 and typical values below 5; this acidity supports barrier enzyme activity and commensal microbiota stability [12]. pH may lag behind TEWL in detecting early barrier normalization over a 12-week horizon. The data suggest that TEWL is the more responsive short-term marker of barrier recovery during TNF-α inhibition, while pH may be better suited for longer-term remodeling or for interventions that directly modulate epidermal lipid processing. Also, it is emphasized that pH findings in psoriasis vary by anatomical site and measurement protocol, underscoring the need to standardize environmental conditions and timing when assessing skin pH in vivo [1].

Although all regions improved, absolute magnitudes varied by site, consistent with the known site-dependent TEWL differences (e.g., higher baseline variability on scalp and back due to hair bearing and mobility). The consistent directionality across sites argues for a class-wide barrier effect with TNF-α blockades, but future work could stratify responses by plaque thickness, scaling, or anatomical biomechanics to refine expectations at specific body areas.

The research strengths include the paired within-subject analyses, multi-site barrier assessments (TEWL, pH), and concurrent clinical/PRO endpoints over a clinically meaningful 12-week interval. However, this study has several important limitations. First, the small sample size (*n* = 9) and single-arm design without a comparator group limit the statistical power and generalizability. The small cohort reflects the exploratory pilot nature of this research; however, the consistent trends across all body sites support the robustness of the observed direction. Second, the absence of a control or comparator group precludes a definitive attribution of the observed TEWL reductions solely to adalimumab therapy. As this was a single-arm pilot study, the findings should therefore be interpreted as exploratory and hypothesis-generating. Third, environmental conditions such as room temperature and relative humidity varied between measurement sessions. Because TEWL and pH are highly sensitive to these parameters, this represents a potential source of measurement variability. Future studies should maintain and report constant conditions (temperature, humidity, acclimatization period, and time of day) and use predefined replicate-reading protocols to enhance reproducibility. Fourth, the measurement protocol did not include formal blinding or inter-rater verification. Although all readings were obtained by a single trained investigator to maintain procedural consistency, future work should incorporate blinded assessments to strengthen reliability and reduce potential operator bias. Fifth, the 12-week follow-up period captures only short-term treatment effects and may not be sufficient to determine whether barrier improvements are sustained over longer durations. Future longitudinal studies with extended follow-ups (≥24–52 weeks) are needed to assess the durability of TEWL normalization and to evaluate potential late-phase changes in skin pH and lipid composition. Finally, while McNemar testing was applied to characterize lesional-to-non-lesional transitions by site, the study was not powered to detect modest categorical shifts; larger controlled cohorts are needed to confirm whether early lesion conversion predicts long-term clinical persistence.

Prospective, controlled comparisons across biologic classes (TNF-α vs. IL-17 vs IL-23) are warranted to determine whether the trajectory and magnitude of TEWL normalization differ by mechanisms of action. Extending follow-ups beyond 12 weeks could assess the durability and potential convergence of pH to physiologic values. Incorporating complementary readouts—stratum–corneum hydration, lipidomics (ceramide subclasses), tight-junction markers, and tape-stripping transcriptomics—would help confirm the observed TEWL changes.

## 5. Conclusions

Twelve weeks of TNF-α inhibitor therapy (adalimumab) in adults with moderate-to-severe plaque psoriasis was associated with a clinically meaningful improvement (PASI, BSA, DLQI) and a consistent reduction in TEWL across all sampled regions (elbow, lower leg, abdomen, back, scalp), suggesting a recovery of the epidermal barrier integrity at lesional sites. In contrast, the skin surface pH remained within a narrow acidic range and showed no significant change, suggesting that TEWL is more responsive short-term barrier metric under TNF-α blockades. These findings indicate that TEWL may serve as a sensitive, objective adjunct to clinical indices when monitoring treatment responses. Given the small sample size, single-arm design, and variation in ambient measurement conditions between visits, the results should be interpreted as exploratory and hypothesis-generating. Larger controlled studies—ideally comparing biologic classes and employing standardized environmental controls—are needed to confirm the magnitude, time course, and anatomical heterogeneity of barrier normalization in psoriasis.

## Figures and Tables

**Table 1:** Comparison of TEWL and pH before and during the treatment with adalimumab.

Body Site	TEWL ± SD (g/m^2^/h) at Baseline	TEWL ± SD (g/m^2^/h) After 12 Weeks	*p*-Value	pH ± SD at Baseline	pH ± SD After 12 Weeks	*p*-Value
Elbow	23.222 ± 7.886	12.167 ± 3.964	0.008	5.061 ± 0.403	5.102 ± 0.314	0.859
Lower leg	21.833 ± 8.653	13.655 ± 7.342	0.008	5.291 ± 0.439	5.162 ± 0.340	0.374
Abdomen	32.589 ± 20.608	13.367 ± 7.497	0.015	5.334 ± 0.421	5.411 ± 0.370	0.594
Scalp	25.611 ± 20.718	11.045 ± 2.581	0.015	5.222 ± 0.461	5.152 ± 0.451	0.767
Back	31.833 ± 24.953	9.589 ± 1.841	0.008	5.146 ± 0.334	5.310 ± 0.376	0.173

## Data Availability

The raw data supporting the conclusions of this article will be made available by the authors on request.

## Abbreviations

The following abbreviations are used in this manuscript:

TEWL	Transepidermal water loss
BSA	Body Surface Area
PASI	Psoriasis Area and Severity Index
TNF-α	Tumor necrosis factor-α
s/c	Subcutaneous
DLQI	Dermatology Life Quality Index
